# Apolipoprotein M (ApoM) Ameliorates Acute Alcohol Intoxication (AAI)‐Hemorrhagic Shock and Resuscitation (HSR)‐Induced Microvascular Leakage

**DOI:** 10.1111/micc.70030

**Published:** 2025-10-10

**Authors:** Mengmeng Chang, Jerome W. Breslin

**Affiliations:** ^1^ Department of Molecular Pharmacology and Physiology, Morsani College of Medicine University of South Florida Tampa Florida USA

**Keywords:** ApoM, endothelial barrier, hemorrhagic shock, junctions, S1P

## Abstract

**Objective:**

Microvascular hyperpermeability is a serious complication that occurs from hemorrhagic shock and resuscitation (HSR), especially when combined with acute alcohol intoxication (AAI). We tested the hypothesis that administration of Apolipoprotein M (ApoM), a lipocalin that normally resides in plasma high‐density lipoproteins (HDL) and a carrier of sphingosine‐1‐phosphate (S1P), reduces combined AAI and HSR‐induced microvascular leakage.

**Methods:**

An established rat model of AAI/HSR was combined with intravital microscopy to study whether the administration of ApoM in resuscitative fluids reduces microvascular leakage of FITC‐albumin. The impact of ApoM on human umbilical vein endothelial cell (HUVEC) monolayer barrier function and junctional integrity was tested, using trans‐endothelial electrical resistance (TER) and immunofluorescence labeling of junctional VE‐Cadherin, respectively. Immunoprecipitation of ApoM in HUVEC and mass spectrometry of complexes were used to determine potential binding partners. The Rac1 G‐LISA assay was used to determine if ApoM causes Rac1 activation in HUVEC.

**Results:**

Compared to sham controls, combined AAI and HSR significantly increased microvascular leakage. Administration of S1P, ApoM, or their combination during resuscitation significantly decreased microvascular leakage. In HUVEC monolayers, with or without alcohol pretreatment, S1P, ApoM, and S1P + ApoM all significantly increased barrier function and improved the junctional integrity of VE‐cadherin compromised by alcohol. The small GTPase Rac1 was found to bind with ApoM in HUVEC and was significantly activated within 5 min of ApoM addition.

**Conclusions:**

The findings suggest that fluid resuscitation with ApoM ameliorates AAI/HSR‐induced microvascular leakage. The mechanism involves stabilizing VE‐Cadherin junction integrity, which could be caused by Rac1 activation.

## 1 Introduction

Traumatic injury is one of the most significant yet often neglected global health concerns. It ranks as one of the leading causes of disability and death, resulting in approximately 4.4 million annual deaths or 8% of total global annual mortality [1, 2]. Major trauma can result in substantial, life‐threatening blood loss, leading to hemorrhagic shock, a primary cause of death in approximately one‐third of admitted trauma patients [3]. A defining characteristic of hemorrhagic shock is systemic microvascular hyperpermeability to plasma proteins, making fluid resuscitation challenging. The initial resuscitation with crystalloid fluids often fails to improve patient outcomes and may even exacerbate microvascular leakage due to its negative impact on plasma oncotic pressure [4, 5]. The use of whole blood or blood products for resuscitation is considered superior [6]; however, the issue of fluid resuscitation remains controversial as strategies aimed at restoring plasma volume following hemorrhagic shock remain suboptimal.

Alcohol intake has been identified as a significant contributing factor in trauma‐related deaths, and has been reported to be present in up to 70% of fatally injured drivers [7]. In the U.S., binge‐drinking results in an estimated $223.5 billion societal cost burden, contributing not only to motor vehicle accidents, but also to interpersonal violence, homicides, and suicides [8, 9, 10]. Up to 80% of trauma patients with alcohol intoxication have been reported to present with hemorrhagic shock. These patients are significantly more hypotensive, require larger resuscitation fluid volumes, and experience poorer outcomes [11, 12, 13]. However, the mechanisms by which alcohol exacerbates trauma outcomes remain poorly understood.

In our previous studies, we demonstrated that hemorrhagic shock and resuscitation (HSR) induces microvascular leakage in the rat mesentery and that acute alcohol intoxication (AAI) worsens HSR‐induced microvascular hyperpermeability [14, 15]. We further showed that fluid resuscitation containing sphingosine‐1‐phosphate (S1P) reduced HSR‐induced microvascular leakage in rats [16]. S1P, a bioactive lipid molecule and phosphorylated metabolite of D‐sphingosine, is derived from multiple cellular sources [17, 18, 19, 20]. It binds to five G‐protein‐coupled receptors and has pleiotropic biological actions [21, 22, 23, 24, 25, 26], including vascular maturation, endothelial cell migration, angiogenesis, and maintenance of barrier function [27, 28, 29, 30]. Extracellular S1P in the circulation is predominantly associated with albumin (~30%) or apolipoprotein M (ApoM, ~65%) [23, 25, 31]. Notably, ApoM is considered the primary carrier of S1P.

ApoM, first identified in 1999 by Xu et al., is a 25‐kDa lipocalin predominantly found in the plasma HDL fraction [32]. The crystal structure of ApoM revealed a typical lipocalin fold with an eight‐stranded antiparallel β‐barrel enclosing an internal binding pocket, likely facilitating the association with small lipophilic ligands such as S1P [33, 34]. Since its discovery, numerous studies have expanded our understanding of the biology and functionality of ApoM, increasing interest in its role. Key findings suggest that the ApoM/S1P axis reduces inflammation, limits monocyte adhesion to the endothelium, reduces the effects of tumor necrosis factor‐α (TNF‐α) on gene expression, and preserves the endothelial barrier [35]. Furthermore, ApoM has been suggested to have cardioprotective properties in animal models [36], and reduced levels of circulating ApoM are independently associated with adverse outcomes in human heart failure [37]. Additionally, the ApoM/S1P axis plays a critical role in lipoprotein metabolism, lipid disorders, atherosclerosis, and endothelial function remodeling [31, 38, 39, 40, 41], likely representing the key physiological functions of ApoM in vivo. However, the specific biological functions of ApoM remain insufficiently explored.

In this study, we initially were curious about whether administration of ApoM could enhance the ability of S1P to reduce microvascular hyperpermeability in an established AAI/HSR rat model. An exciting finding was that administration of ApoM without exogenous S1P significantly ameliorated AAI/HSR‐induced microvascular permeability. This led us to investigate more deeply the role of ApoM by itself in AAI/HSR‐induced microvascular leakage.

## 2 Methods

### 2.1 Animals

All animal procedures were conducted in strict adherence to the U.S. Animal Welfare Act, the U.S. Public Health Service Policy on the Humane Care and Use of Laboratory Animals, and the NIH *Guide for the Care and Use of Laboratory Animals*. All experiments were approved by the Institutional Animal Care and Use Committee (IACUC) at the University of South Florida under protocol IS00008866. Both male and female Sprague–Dawley rats (12–16 weeks old, weighing 330–355 g) were procured from Inotiv (Indianapolis, IN). The animals were housed in a controlled vivarium environment (22°C, 12‐h light/dark cycle) and provided a standard diet (Purina Rat Chow, Ralston Purina) along with ad libitum access to water. The rats were allowed a one‐week acclimation period before undergoing surgery. All animals were humanely euthanized with Somnasol Euthanasia‐III Solution (87 mg/kg, i.v., Henry Schein, Dublin, OH) in accordance with the experimental protocols.

### 2.2 Surgical Preparation

The surgical preparation for catheter implantation has been previously described in detail [15, 16, 42]. Briefly, rats were anesthetized with isoflurane (2%–3% induction, 1%–2% maintenance). Prior to surgery, rats received an injection of Meloxicam (4 mg/kg) as a preemptive long‐acting (72 h) analgesic for potential surgical pain. Sterile catheters, pre‐flushed with 0.9% sterile sodium chloride USP (Baxter, Deerfield, IL), were implanted into the left common carotid artery, the right external jugular vein, and the stomach. All catheters were plugged and sealed and routed subcutaneously using a trocar to the dorsal nape of the neck. The catheters were then secured to the closed incision with suture, coiled, and wrapped with tape to prevent any damage. The carotid catheter was used for blood pressure monitoring and blood sample collection, while the jugular catheter was employed for the administration of resuscitation fluids and fluorophore tracers for intravital microscopy. The gastric catheter was used for alcohol administration. Following surgery, the animals were housed individually for a recovery period of 3–4 days prior to experiments.

### 2.3 Alcohol Administration and the Fixed‐Pressure HSR Protocol

The combined acute alcohol intoxication (AAI) and HSR protocol in conscious, unrestrained rats was performed similarly to that previously described [15, 16, 42, 43, 44]. Briefly, the rats were placed in small cages, and the previously implanted catheters were routed through the tops of the cages. The carotid catheter was connected to a pressure transducer for continuous blood pressure monitoring using an ADInstruments PowerLab 4/35 system with a Quad Bridge amplifier and LabChart software (ADInstruments, Colorado Springs, CO).

The rats were given at least 30 min to acclimate to their cages in order to minimize the impact of handling‐induced stress on the experiments. Next, blood pressure was recorded for 30 min to determine baseline. Following the baseline measurements, a bolus of 30% alcohol (2.5 g/kg ethanol; Pharmco by Greenfield Global, Brookfield, CT) was administered via the gastric catheter to simulate a binge‐drinking episode as previously described [15, 45, 46]. Sham, control rats received isovolumic water. Following alcohol administration, the rats were allowed to roam freely in their cages with continuous monitoring of blood pressure for 30 min before experimental hemorrhage. To initiate hemorrhagic shock, arterial blood was withdrawn to reduce mean arterial pressure (MAP), which was maintained at around 40–60 mmHg for 60 min. Small additional volumes of blood were drawn if needed to keep the pressure near 40 mmHg. At the conclusion of the hemorrhage period, warm (37°C) Lactated Ringer's solution was administered intravenously. First, a bolus equal to 40% of the total blood removed (TBR) was administered, and this was followed by a continuous infusion of Lactated Ringer's solution equal to two times the TBR over a 60‐min period. Stock solutions containing S1P (Tocris, Minneapolis, MN), ApoM (MyBioSource, San Diego, CA), or their combination were dissolved into the Lactated Ringer's solution before resuscitation was performed. The final doses were 0.1 mg/kg for S1P and 0.4 mg/kg for ApoM. Our previous studies demonstrated that this dose of S1P significantly reduces microvascular leakage [15, 47]. Additionally, the normal range of ApoM in human plasma has been reported to be 0.6–1.3 μM [48]. The dose of ApoM in our study was calculated to maintain the plasma concentration around 1 μM during fluid resuscitation.

### 2.4 In Vivo Assessment of Microvascular Leakage

Immediately after the AAI/HSR protocol, microvascular leakage of FITC‐albumin from the mesenteric microcirculation was assessed using intravital microscopy (IVM) as previously described [15, 16, 49, 50, 51]. Briefly, the rats were anesthetized with isoflurane (2%–3% induction, 1%–2% maintenance). Body temperature was maintained with a rodent heating pad throughout the protocol. The abdomen was shaved, and a midline laparotomy was performed. A loop of small intestine and mesentery was carefully exteriorized and positioned over an optical stage. The mesentery was superfused with 37°C Ringer's solution. Blood pressure was monitored via the carotid catheter connected to the pressure transducer. FITC‐conjugated albumin (Sigma‐Aldrich, St. Louis, MO) dissolved in Lactated Ringer's solution was administered as a bolus via the jugular vein (0.1 mg/kg over 1–2 min), followed by continuous infusion (0.15 mg/kg/min) to maintain a steady‐state plasma concentration. The mesenteric microcirculation was observed using a fluorescent microscope (Nikon Eclipse E600) with a 4× objective (Nikon Instruments Inc., Natick, MA) at 488 nm excitation. Images were captured at 10‐min intervals over a 30‐min period with a Photometrics CoolSnap HQ2 camera (Teledyne Photometrics, Tucson, AZ) controlled by Micromanager (NIH ImageJ) software. The integrated optical intensity (IOI) of extravascular regions near postcapillary venules was measured to assess the degree of FITC‐albumin extravasation as previously described [52].

### 2.5 Endothelial Cell Culture

Pooled human umbilical vein endothelial cells (HUVEC) were purchased from LifeLine Cell Technology (Frederick, MD) and cultured in Vasculife endothelial cell culture growth medium (VGM, Lifeline) on a 1.5% porcine gelatin matrix (Sigma‐Aldrich, St. Louis, MO) in a 37°C, 5% CO_2_ incubator as previously described [53]. On the day of the experiment, the growth medium was replaced with serum‐free Vasculife basal medium (VBM). For experiments, HUVEC were used between passages 2 and 5. The justification for utilizing HUVEC is that, as their name implies, they are primary cells isolated from the vein of the umbilical cord, and they have been used as a foundational tool in cell biology research to study endothelial cells in vitro due to their ease of isolation and cultivation. In addition, HUVEC provided nearly identical results compared to microvascular endothelial cells when treated with alcohol or S1P [14, 54].

### 2.6 Determination of Endothelial Barrier Function

Confluent HUVEC monolayers served as an in vitro model to evaluate barrier function, assessed using an Electric Cell/Substrate Impedance Sensing (ECIS) ΖΘ system (Applied Biophysics, Troy, NY) [14] were grown to confluence in VGM on gelatin‐coated, gold‐plated 8W1E ECIS electrode arrays. The arrays were then placed on the ECIS station in a 37°C, 5% CO_2_ incubator for overnight monitoring of barrier maturation. Prior to experimentation, the medium was replaced with VBM, and the cells were allowed to stabilize, establishing a steady baseline trans‐endothelial electrical resistance (TER) between 8000 and 12,000 Ω for at least 1–2 h. After the baseline period, cells were treated with either 0.1 μM S1P, 1 μM ApoM, or their combination (1 h) to test potential impacts on TER in the absence or presence of 75 mM alcohol (10 min). Alcohol applied at 75 mM was previously determined as optimal for disrupting barrier function of cultured endothelial monolayers [47]. 0.1 μM S1P was chosen as it was previously shown to significantly elevate barrier function [15, 54]. ApoM was applied at 1 μM based upon the normal plasma concentration range found in humans [48].

### 2.7 Immunofluorescence Labeling and Confocal Microscopy

HUVEC were grown to confluence in VGM on round glass #1 coverslips coated with 1.5% porcine gelatin in 6‐well plates. The confluent cell monolayers were then incubated for 2 h in VBM to alleviate potential confounding factors caused by serum prior to treatment with 75 mM alcohol (5 min) followed by the absence or presence of 0.1 μM S1P, 1 μM ApoM, or combined S1P and ApoM (1 h). Immunofluorescence labeling and confocal microscopy were performed as previously described [14, 55, 56]. Briefly, the cells were fixed with 4% paraformaldehyde for 10 min at room temperature, followed by two 3‐min washes with 100 mM glycine buffer to quench the fixation, and then one 3‐min wash with Ca^2+^/Mg^2+^‐free Dulbecco's PBS (DPBS). The cells were then permeabilized with 0.1% TritonX‐100 in 1X PBS for 10 min and followed by three 5‐min washes with DPBS to remove the TritonX‐100 detergent. After permeabilization, cells were blocked in 5% donkey serum in DPBS for 60 min and then incubated overnight at 4°C with rabbit anti‐VE‐Cadherin primary antibody (ThermoFisher, Waltham, MA, cat. # 36‐1900), diluted 1:25 in 1% donkey serum. After overnight incubation with primary antibody, the cells were rinsed with three 10‐min washes in antibody wash solution (151 mM NaCl, 17 mM trisodium citrate, and 0.05% Triton X‐100) and then incubated with an Alexafluor‐488 conjugated donkey anti‐rabbit IgG secondary antibody (Invitrogen/ThermoFisher; A21206) diluted 1:100 in 1% donkey serum solution for 60 min at room temperature, followed by three 10‐min rinses in antibody wash solution. After the last wash, the coverslips with cells were removed from the wells and mounted face down Vectashield (Vector Laboratories, Newark, CA) on glass microscope slides. Confocal images were acquired with a Leica SP8 laser confocal microscope with 20X objective at the Lisa Muma Weitz Advanced Microscopy and Cell Imaging Core at the University of South Florida. The images were analyzed using ImageJ software.

### 2.8 Proteomics

A proteomics approach was used to find potential cellular proteins that may form complexes with exogenous ApoM. HUVEC were grown to confluence in 6‐well plates. After a 2‐h incubation in serum‐free medium (VBM), the cells were either left untreated or treated with 1 μM ApoM for 1 h. The cells were washed twice with ice‐cold DPBS and then lysed with ice‐cold RIPA buffer (Millipore Sigma, Burlington, MA) containing HALT protease inhibitor cocktail (ThermoFisher). The lysates were centrifuged at 14,000 × g for 10 min, the supernatants collected, and mouse anti‐ApoM antibody (Cell Signaling Technology, Danvers, MA, cat. # 5709S) was added at a 1:50 dilution, followed by overnight incubation on a rocker at 4°C. The sample was diluted in Tris‐buffered saline (TBS) solution containing 0.05% Tween‐20 detergent (wash buffer) and Protein A/G magnetic beads (ThermoFisher PI88802) were added to bind to the mouse anti‐ApoM antibodies. After incubation for 1 h on a rocker at room temperature, the mixture was transferred to a magnetic stand, washed twice with wash buffer and once with MilliQ water, and then eluted with 0.1 M glycine, pH 2.0, and neutralized with 1 M Tris, pH 7.5. The immunoprecipitated samples were then brought to the USF Health Proteomics Core Facility for STRAP digestion followed by characterization using a Thermo Q‐exactive‐HF mass spectrometer coupled to a Thermo Easy nLC 1200. The mass spectrometric data were processed using Proteome Discoverer (ThermoFisher). The reviewed human database was downloaded from Uniprot and searched with the following parameters: tryptic enzyme with a max of 2 missed cleavages, a precursor mass tolerance of 10 ppm and a fragment mass tolerance of 0.02 Da. Modifications included oxidation, acetyl, and carbamidomethyl. The FDR rate was set at 0.01, and LFQ intensities were compared between samples for ID.

### 2.9 Rac1 Activation Assay

Activated, GTP‐bound Rac1 was detected and quantified using the Rac1 G‐LISA activation assay (Cytoskeleton Inc., Denver, CO, catalog #BK126). HUVEC were grown in 10‐cm culture dishes to confluence. The growth medium (VGM) was changed to VBM for at least 2 h prior to experiments to alleviate potential confounding effects of serum. The cells were treated with 1 μM ApoM for 2, 5, 10, or 30 min before being rinsed with ice‐cold PBS. Ice‐cold lysis buffer containing protease inhibitors was added, and the cells were quickly scraped and harvested, which was snap‐frozen immediately in liquid nitrogen. The Precision Red Advanced Protein Assay (Cytoskeleton Inc.) and a SpectraMax M3 plate reader (Molecular Devices, Sunnyvale, CA) were used to quantify protein concentration. Protein concentrations were then equalized among the samples for the assay. GTP‐bound levels of Rac1 were determined in a 96‐well Rac1‐GTP binding plate. Wells containing lysis buffer only served as a negative control and with 80 pg/mL recombinant GTP‐bound Rac1 as a positive control. Luminescence was determined with the SpectraMax M3 plate reader.

### 2.10 Data Analyses

Data is presented as means ± SEM. GraphPad Prism 10 software was used for analysis and preparation of figures. Multi‐group comparisons were performed using one‐way or two‐way ANOVA, followed by Tukey's or Sidak's post hoc test to perform multiple comparisons. Significance was accepted at *p* < 0.05.

## 3 Results

### 3.1 ApoM Ameliorates AAI/HSR‐Induced Mesenteric Microvascular Leakage

**FIGURE 1:**
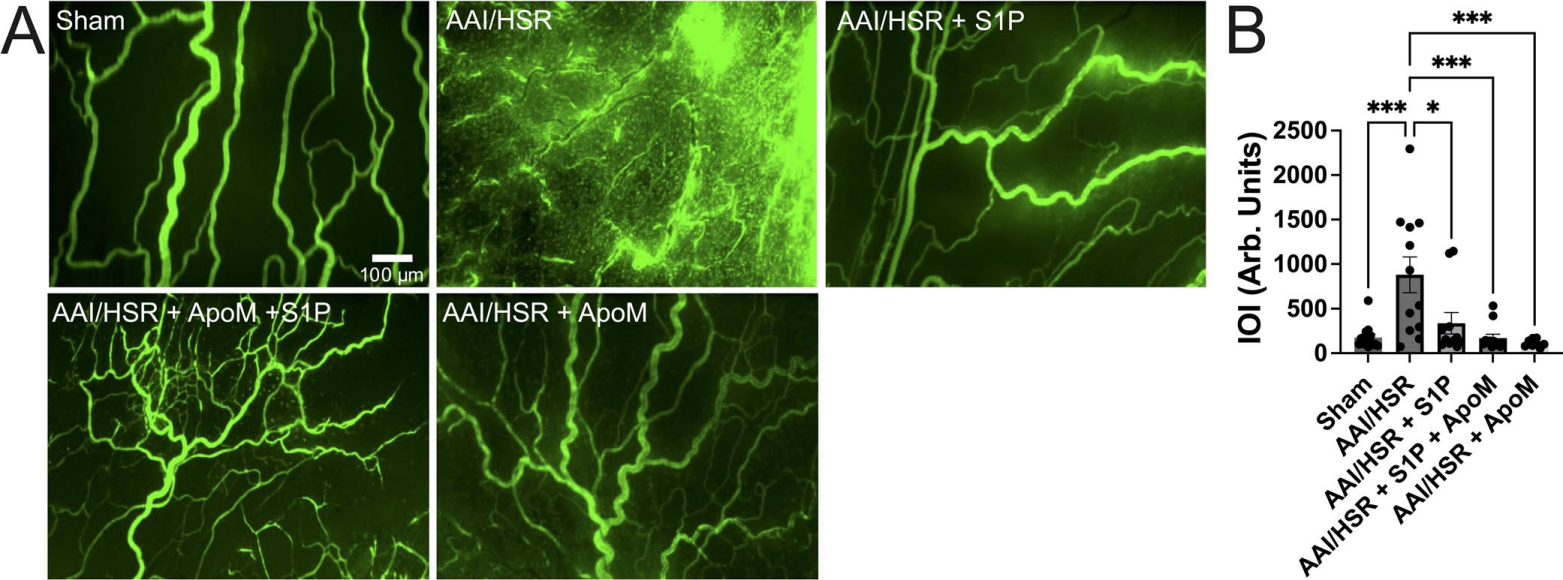
ApoM reduces AAI/HSR‐induced microvascular leakage of plasma proteins. (A) Representative intravital microscopy images. (B) Summarized data. Each group had both male and female rats. *p < 0.05 and ***p < 0.001 between groups. One‐way ANOVA followed by Tukey's test. The number of rats in each group was: Sham, *N* = 13 (seven male, six female); AAI/HSR, *N* = 12 (six male, six female); AAI/HSR + S1P, *N* = 11 (six male, five female); AAI/HSR + S1P + ApoM, *N* = 12 (six male, six female); AAI/HSR + ApoM, *N* = 8 (six male, two female).

As we previously observed that administration of S1P in fluid resuscitation can reduce AAI/HSR‐induced microvascular leakage, and ApoM is considered the main carrier of S1P in plasma, we evaluated if adding ApoM to resuscitation fluids impacts AAI/HSR‐induced microvascular leakage. Figure 1A shows representative intravital microscopy images and Figure 1B contains the summarized IOI data for each experimental group. In the sham group, intravenously administered FITC‐albumin remained in the luminal compartment of the microvessels, with minimal fluorescence intensity in the extravascular space, resulting in relatively low IOI values. In contrast, rats that underwent AAI/HSR showed high levels of microvascular leakage of FITC‐albumin, evidenced by the high fluorescence intensity in the areas surrounding the mesenteric microvasculature, raising the IOI. In a similar fashion to what we observed in our previous study [15], the addition of S1P to the resuscitation fluid reduced the AAI/HSR‐induced increase in IOI significantly. The addition of S1P plus ApoM in the resuscitation fluid also significantly reduced the IOI compared to AAI/HSR alone. Lastly, AAI/HSR rats that received only ApoM in their resuscitation fluid also had significantly lower IOI levels compared to the AAI/HSR group. These data confirm the ability of S1P administration for ameliorating AAI/HSR‐induced microvascular leakage of plasma proteins but also suggest that ApoM administration alone attenuates the microvascular hyperpermeability induced by AAI/HSR.

### 3.2 ApoM Enhances Barrier Function of HUVEC Monolayers

**FIGURE 2:**
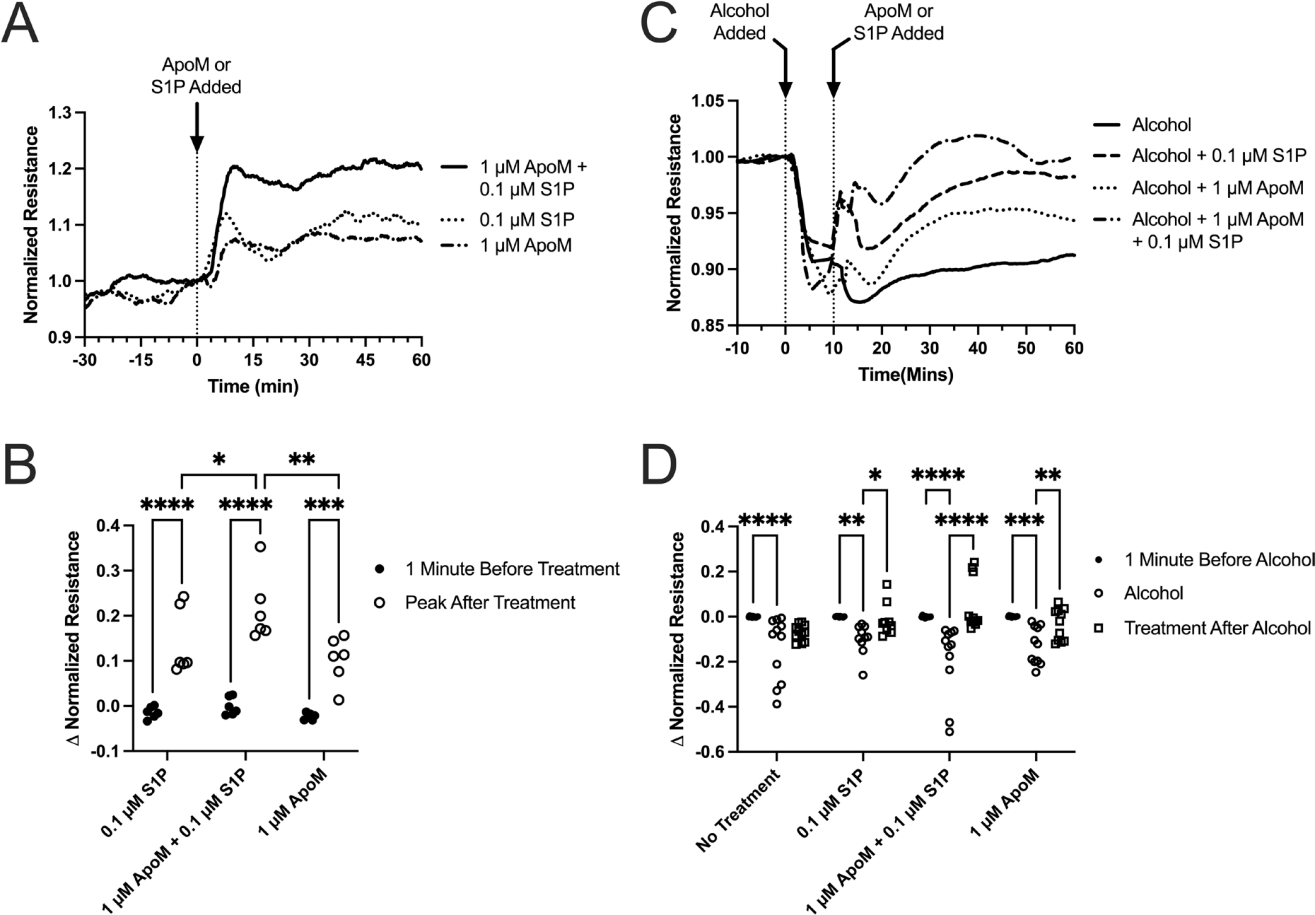
ApoM enhances barrier function of HUVEC monolayers. (A) Traces of mean normalized TER over time, before and after the addition of ApoM, S1P, or combined ApoM + S1P. Resistance is normalized to time = 0 min at the time of addition of the treatments. (B) Summarized data, showing the peak change in TER within 15 min of treatment, compared to baseline (recorded 1 min before the addition of treatments) for each group. *N* = 6 independent experiments for each group. (C) Traces of mean normalized TER over time, before and after the addition of alcohol (75 mM), followed by treatment with ApoM (*N* = 11), S1P (*N* = 10), or combined ApoM + S1P (*N* = 11), or no treatment (*N* = 11). Resistance is normalized to time = 0 min at the time of addition of alcohol. (D) Summarized data, showing the TER peak decreases in response to alcohol in each group (within the first 7–10 min), and the TER peak changes after the addition of ApoM, S1P, or ApoM + S1P, or no treatment. For panels (C and D), *N* indicates the total number of replicates for each group. *p < 0.05, **p < 0.01, ***p < 0.001, ****p < 0.0001 between groups. Two‐way ANOVA and Sidak's test.

To better understand how ApoM improves the microvascular hyperpermeability caused by AAI/HSR, we utilized an in vitro approach to isolate the direct impact of ApoM on endothelial cells. For this, cultured HUVEC monolayers served as a model of the endothelial barrier in ECIS, which is a real‐time, label‐free, impedance‐based approach to study the barrier dynamics [57, 58], was used to test the direct impact of ApoM on endothelial barrier function. HUVEC monolayers were treated with 1 μM ApoM, 0.1 μM S1P, or combined 1 μM ApoM + 0.1 μM S1P. The time courses of mean TER, an index of barrier integrity, were recorded. The traces show that the TER was noticeably increased after ApoM or S1P were added and that the combination of ApoM + S1P produced an even greater increase in TER (Figure 2A). When comparing the mean TER before and after the addition of S1P, ApoM, or their combination, all these treatments caused a significant increase in TER (Figure 2B). In addition, the combination of ApoM and S1P increased the TER significantly more than ApoM or S1P alone (Figure 2B). These data suggest that ApoM, when added alone, enhances endothelial barrier function of HUVEC monolayers, and when added together with S1P, generates an even stronger barrier protective effect. We also repeated this experiment after challenging the HUVEC monolayers with 75 mM alcohol for up to 10 min, which caused a mild, yet significant decrease in TER (Figure 2C). Addition of 1 μM ApoM, 0.1 μM S1P, or combined 1 μM ApoM + 0.1 μM S1P ameliorated the alcohol‐induced disruption of the barrier function (Figure 2D).

### 3.3 ApoM Ameliorates Alcohol‐Induced Compromised VE‐Cadherin Integrity in HUVEC Monolayers

**FIGURE 3:**
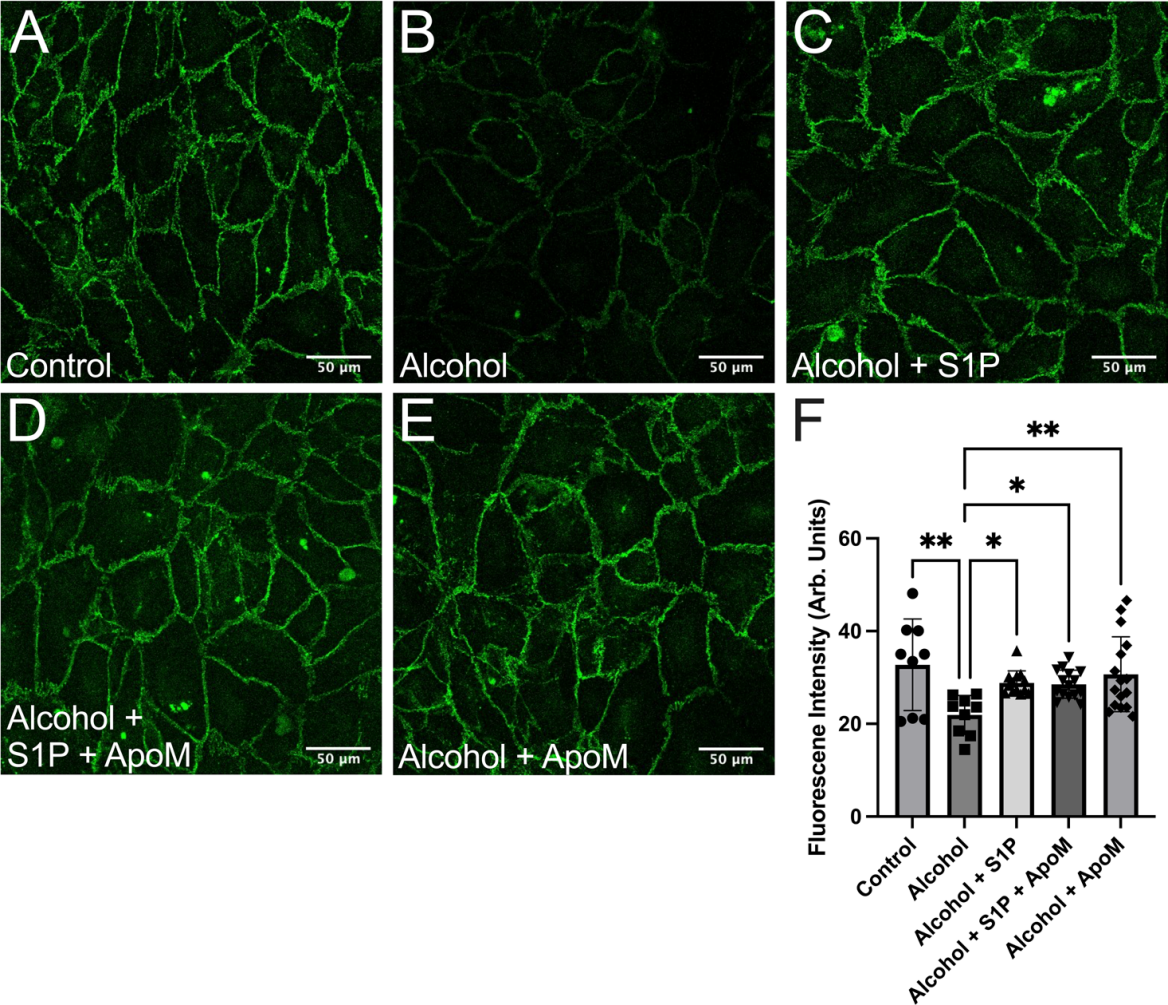
ApoM and S1P ameliorate alcohol‐induced loss of junctional VE‐cadherin in HUVEC monolayers. Representative confocal images of immunolabeling of VE‐cadherin (green) for control (A), alcohol‐treated cells (B), cells treated with alcohol followed by S1P (C), alcohol followed by S1P and ApoM (D), and cells treated with alcohol followed by ApoM alone (E). Panel (F) shows the summarized data of the intensity of VE‐cadherin labeling at junctions. *p < 0.05, **p < 0.01 between groups. One‐way ANOVA and Tukey's test. The number of replicates analyzed for each group are: Control, *N* = 9; alcohol, *N* = 9; alcohol + S1P, *N* = 13; alcohol + S1P + ApoM, *N* = 15; alcohol + ApoM alone, *N* = 16. The replicates were obtained from 3 independent experiments.

Studies modeling alcohol‐induced brain endothelial barrier dysfunction showed that tight junction proteins were disrupted [59, 60, 61], while our previous studies found that alcohol disrupts VE‐Cadherin organization at intercellular junctions [14]. VE‐Cadherin is an endothelial‐specific junctional protein, playing a critical role in peripheral microvascular barrier function [62]. We aimed to investigate whether ApoM enhances endothelial barrier function by improving VE‐Cadherin integrity using immunofluorescence confocal microscopy. To mimic AAI in vitro, we utilized 75 mM alcohol to disrupt normal VE‐Cadherin organization at junctions prior to incubation of S1P, ApoM, or combined ApoM and S1P. Figure 3A shows that VE‐Cadherins localize at cell–cell junctions in control HUVEC, displaying a continuous and clear boundary. Alcohol significantly decreased the apparent intensity of VE‐Cadherin labeling (Figure 3B), which was improved by treatment with S1P (Figure 3C), S1P combined with ApoM (Figure 3D), or ApoM alone (Figure 3E). The quantified intensities of VE‐Cadherin labeling at cell–cell junctions shown in Figure 3F suggest that S1P, ApoM + S1P, and ApoM alone, when applied after alcohol treatment, all significantly rescued the alcohol‐induced compromise of endothelial adherens junctions.

### 3.4 Rac1 Is Involved in ApoM‐Mediated Signaling

**FIGURE 4:**
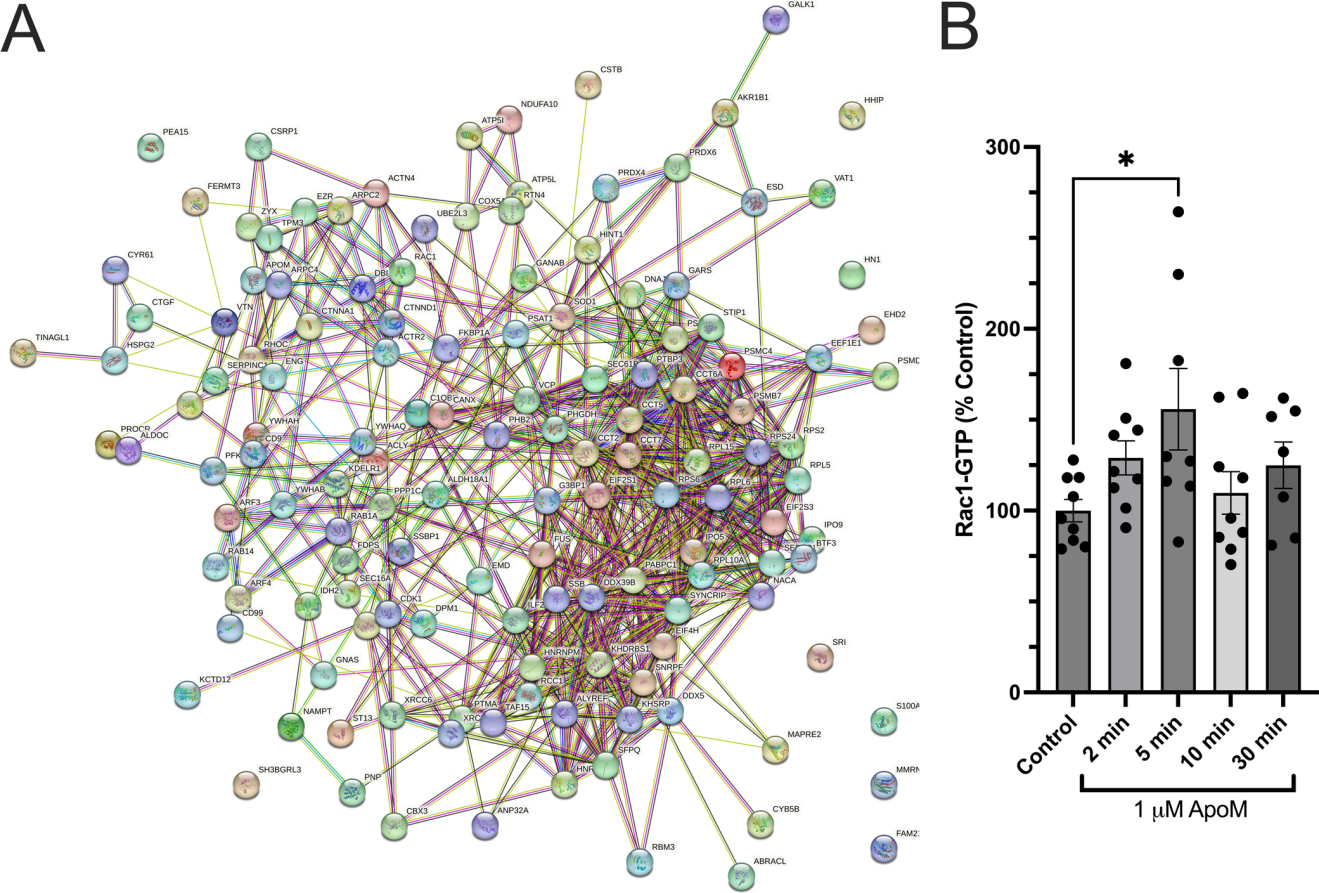
Detection of Rac1 in an ApoM pull‐down assay and confirmation of Rac1 activation in HUVEC treated with ApoM. (A) String‐DB diagram of all proteins detected in the ApoM immunoprecipitate from ApoM‐treated HUVEC lysate. These proteins are also listed in Table 1. (B) Rac1‐GTP detected in control HUVEC and those treated with 1 μM ApoM at the indicated time points. *p < 0.05 compared to control. One‐way ANOVA followed by Dunnett's test. The number of replicates for each group is: Control, *N* = 9; ApoM 2 min, *N* = 9; ApoM 5 min, *N* = 8; ApoM 10 min, *N* = 9; ApoM 30 min, *N* = 7. Each replicate represents an individual dish of cultured HUVEC, obtained from five independent experiments.

The precise role of ApoM as part of an S1P/ApoM axis or as an activator of endothelial signaling on its own has not been thoroughly investigated. To explore the potential function of ApoM by itself (not pre‐conjugated to S1P), confluent HUVEC monolayers were treated with 1 μM ApoM for 1 h. Afterward, the cell lysates were obtained, and immunoprecipitation of ApoM was carried out to identify what proteins may form complexes with ApoM. Mass spec analysis revealed 139 potential binding partners that were pulled down with ApoM in HUVEC lysate (Table 1). The network diagram generated for this group of proteins is shown in Figure 4A. The 139 nodes are connected with 702 edges, with a protein–protein enrichment *p*‐value < 10^−16^, significantly more edges than expected for a random set of proteins of the same size and degree distribution from the human genome. Analysis of the network revealed protein network enrichment in proteins localized to cell‐substrate junctions, anchoring junctions, cell junctions, and the Arp2/3 complex (Table 2). One key protein of interest found in these enriched pathways/functions in this list is Rac1, which plays a critical role in maintaining and stabilizing endothelial barrier function through regulation of VE‐Cadherin [63, 64, 65, 66, 67, 68]. As shown in Figure 3, ApoM ameliorates the junctional loss of VE‐cadherin caused by alcohol exposure, so whether ApoM causes activation of Rac1 in cultured HUVEC monolayers was investigated. We evaluated the time course of Rac1 activation by ApoM using the Rac1‐GLISA assay that detects Rac1 in its GTP‐bound active form. The results show that Rac1 is significantly activated at the 5‐min time point after ApoM addition (Figure 4B). This finding suggests that ApoM elicits responses in HUVEC at least in part through activation of Rac1.

**TABLE 1:** Proteins identified in the ApoM pull‐down mass spectrometry analysis.

UniProt ID	Description	UniProt ID	Description
O95445	Apolipoprotein M (APOM)	P62847	40S ribosomal protein S24 (RPS24)
O43707	Alpha‐actinin‐4 (ACTN4)	P12956	X‐ray repair cross‐complementing protein 6 (XRCC6)
P18085	ADP‐ribosylation factor 4 (ARF4)	P41250	Glycine–tRNA ligase (GARS1)
Q8TD47	40S ribosomal protein S4, Y isoform 2 (RPS4Y2)	Q07666	KH domain‐containing, RNA‐binding, signal transduction‐associated protein 1 (KHDRBS1)
P43490	Nicotinamide phosphoribosyltransferase (NAMPT)	P24390	ER lumen protein‐retaining receptor 1 (KDELR1)
P06753	Tropomyosin alpha‐3 chain (TPM3)	P98160	Basement membrane‐specific heparan sulfate proteoglycan core protein (HSPG2)
P11940	Polyadenylate‐binding protein 1 (PABPC1)	Q86UX7	Fermitin family homolog 3 (FERMT3)
P55072	Transitional endoplasmic reticulum ATPase (VCP)	P53396	ATP‐citrate synthase (ACLY)
P61204	ADP‐ribosylation factor 3 (ARF3)	Q15056	Eukaryotic translation initiation factor 4H (EIF4H)
Q14697	Neutral alpha‐glucosidase AB (GANAB)	Q04917	14‐3‐3 protein eta (YWHAH)
Q13162	Peroxiredoxin‐4 (PRDX4)	P06493	Cyclin‐dependent kinase 1 (CDK1)
P31946	14–3‐3 protein beta/alpha (YWHAB)	Q13185	Chromobox protein homolog 3 (CBX3)
P15311	Ezrin (EZR)	P09972	Fructose‐bisphosphate aldolase C (ALDOC)
P40227	T‐complex protein 1 subunit zeta (CCT6A)	P63000	Ras‐related C3 botulinum toxin substrate 1 (RAC1)
P00491	Purine nucleoside phosphorylase (PNP)	O60716	Catenin delta‐1 (CTNND1)
P27348	14–3‐3 protein theta (YWHAQ)	P04004	Vitronectin (VTN)
P68036	Ubiquitin‐conjugating enzyme E2 L3 (UBE2L3)	P21926	CD9 antigen (CD9)
Q99623	Prohibitin‐2 (PHB2)	O15027	Protein transport protein Sec16A (SEC16A)
P15880	40S ribosomal protein S2 (RPS2)	O43169	Cytochrome b5 type B (CYB5B)
E9PAV3	Nascent polypeptide‐associated complex subunit alpha, muscle‐specific form (NACA)	P62136	Serine/threonine‐protein phosphatase PP1‐alpha catalytic subunit (PPP1CA)
P98179	RNA‐binding protein 3 (RBM3)	Q96CX2	BTB/POZ domain‐containing protein KCTD12 (KCTD12)
P17844	Probable ATP‐dependent RNA helicase DDX5 (DDX5)	O43175	D‐3‐phosphoglycerate dehydrogenase (PHGDH)
P13010	X‐ray repair cross‐complementing protein 5 (XRCC5)	Q99832	T‐complex protein 1 subunit eta (CCT7)
Q13283	Ras GTPase‐activating protein‐binding protein 1 (G3BP1)	P52272	Heterogeneous nuclear ribonucleoprotein M (HNRNPM)
P35637	RNA‐binding protein FUS (FUS)	P59998	Actin‐related protein 2/3 complex subunit 4 (ARPC4)
P14324	Farnesyl pyrophosphate synthase (FDPS)	O75832	26S proteasome non‐ATPase regulatory subunit 10 (PSMD10)
Q96P70	Importin‐9 (IPO9)	P43686	26S proteasome regulatory subunit 6B (PSMC4)
P31948	Stress‐induced‐phosphoprotein 1 (STIP1)	P23246	Splicing factor, proline‐ and glutamine‐rich (SFPQ)
P06454	Prothymosin alpha (PTMA)	Q99436	Proteasome subunit beta type‐7 (PSMB7)
P48643	T‐complex protein 1 subunit epsilon (CCT5)	P00441	Superoxide dismutase [Cu‐Zn] (SOD1)
P30041	Peroxiredoxin‐6 (PRDX6)	Q15121	Astrocytic phosphoprotein PEA‐15 (PEA15)
P20290	Transcription factor BTF3 (BTF3)	P20674	Cytochrome c oxidase subunit 5A, mitochondrial (COX5A)
Q96QV1	Hedgehog‐interacting protein (HHIP)	Q9NQC3	Reticulon‐4 (RTN4)
P50502	Hsc70‐interacting protein (ST13)	Q9UK76	Jupiter microtubule‐associated homolog 1 (JPT1)
P62820	Ras‐related protein Rab‐1A (RAB1A)	O60762	Dolichol‐phosphate mannosyltransferase subunit 1 (DPM1)
P08134	Rho‐related GTP‐binding protein RhoC (RHOC)	P48735	Isocitrate dehydrogenase [NADP], mitochondrial (IDH2)
P07108	Acyl‐CoA‐binding protein (DBI)	Q12905	Interleukin enhancer‐binding factor 2 (ILF2)
P04080	Cystatin‐B (CSTB) 2	Q15555	Microtubule‐associated protein RP/EB family member 2 (MAPRE2)
Q92945	Far upstream element‐binding protein 2 (KHSRP)	O15144	Actin‐related protein 2/3 complex subunit 2 (ARPC2)
Q9P1F3	Costars family protein ABRACL (ABRACL)	P10768	S‐formylglutathione hydrolase (ESD)
O95299	NADH dehydrogenase [ubiquinone] 1 alpha subcomplex subunit 10, mitochondrial (NDUFA10)	P14209	CD99 antigen (CD99)
Q9GZM7	Tubulointerstitial nephritis antigen‐like (TINAGL1)	Q96FQ6	Protein S100‐A16 (S100A16)
P51570	Galactokinase (GALK1)	P49773	Adenosine 5′‐monophosphoramidase HINT1 (HINT1)
P15144	Aminopeptidase N (ANPEP)	Q99536	Synaptic vesicle membrane protein VAT‐1 homolog (VAT1)
P18754	Regulator of chromosome condensation (RCC1)	P17813	Endoglin (ENG)
P62942	Peptidyl‐prolyl cis‐trans isomerase FKBP1A (FKBP1A)	Q02878	60S ribosomal protein L6 (RPL6)
Q9UNN8	Endothelial protein C receptor (PROCR) 1	Q07021	Complement component 1 Q subcomponent‐binding protein, mitochondrial (C1QBP)
P41091	Eukaryotic translation initiation factor 2 subunit 3 (EIF2S3)	P62306	Small nuclear ribonucleoprotein F (SNRPF)
Q15942	Zyxin (ZYX)	P60468	Protein transport protein Sec61 subunit beta (SEC61B)
O43324	Eukaryotic translation elongation factor 1 epsilon‐1 (EEF1E1)	O95758	Polypyrimidine tract‐binding protein 3 (PTBP3)
Q92804	TATA‐binding protein‐associated factor 2 N (TAF15)	P56385	ATP synthase subunit e, mitochondrial (ATP5ME)
Q9H299	SH3 domain‐binding glutamic acid‐rich‐like protein 3 (SH3BGRL3)	P30626	Sorcin (SRI)
O00410	Importin‐5 (IPO5)	O00622	CCN family member 1 (CCN1)
Q13201	Multimerin‐1 (MMRN1)	P61106	Ras‐related protein Rab‐14 (RAB14)
P21291	Cysteine and glycine‐rich protein 1 (CSRP1)	Q13838	Spliceosome RNA helicase DDX39B (DDX39B)
P62753	40S ribosomal protein S6 (RPS6)	P46777	60S ribosomal protein L5 (RPL5)
P01008	Antithrombin‐III (SERPINC1)	P35221	Catenin alpha‐1 (CTNNA1)
P27824	Calnexin (CANX)	Q8NC51	Plasminogen activator inhibitor 1 RNA‐binding protein (SERBP1)
P05198	Eukaryotic translation initiation factor 2 subunit 1 (EIF2S1)	O60506	Heterogeneous nuclear ribonucleoprotein Q (SYNCRIP)
Q9BRX8	Peroxiredoxin‐like 2A (PRXL2A)	Q13200	26S proteasome non‐ATPase regulatory subunit 2 (PSMD2)
P15121	Aldo‐keto reductase family 1 member B1 (AKR1B1)	P54886	Delta‐1‐pyrroline‐5‐carboxylate synthase (ALDH18A1)
Q04837	Single‐stranded DNA‐binding protein, mitochondrial (SSBP1)	Q5JWF2	Guanine nucleotide‐binding protein G(s) subunit alpha isoforms XLas (GNAS)
Q16531	DNA damage‐binding protein 1 (DDB1)	P50402	Emerin (EMD)
Q01813	ATP‐dependent 6‐phosphofructokinase, platelet type (PFKP)	P29279	CCN family member 2 (CCN2)
P62906	60S ribosomal protein L10a (RPL10A)	P39687	Acidic leucine‐rich nuclear phosphoprotein 32 family member A (ANP32A)
Q86V81	THO complex subunit 4 (ALYREF)	P61313	60S ribosomal protein L15 (RPL15)
O75964	ATP synthase subunit g, mitochondrial (ATP5MG)	Q9Y617	Phosphoserine aminotransferase (PSAT1)
Q13151	Heterogeneous nuclear ribonucleoprotein A0 (HNRNPA0)	Q9NZN4	EH domain‐containing protein 2 (EHD2)
P61160	Actin‐related protein 2 (ACTR2)	O60884	DnaJ homolog subfamily A member 2 (DNAJA2)
P78371	T‐complex protein 1 subunit beta (CCT2)	P05455	Lupus La protein (SSB)

**TABLE 2:** Selected GO components enriched in the ApoM pull‐down dataset.

Term ID	Term description	FDR	Matching proteins in your network (labels)
GO:0030055	Cell‐substrate junction	6.52E−12	PROCR, ACTN4, FERMT3, ARPC2, MAPRE2, CTNNA1, PABPC1, ZYX, RPS2, RAC1, EZR, CSRP1, RPL5, YWHAB, ENG, RPL10A, HSPG2, ACTR2, CD99, YWHAQ, CD9, G3BP1, RPL6
GO:0070161	Anchoring junction	3.42E−09	PROCR, YWHAH, ACTN4, FERMT3, AKR1B1, ARPC2, MAPRE2, CTNNA1, PABPC1, ZYX, PPP1CA, RPS2, RAC1, EZR, CSRP1, RPL5, YWHAB, ENG, RPL10A, HSPG2, ACTR2, CD99, YWHAQ, CD9, G3BP1, CTNND1, RPL6
GO:0030054	Cell junction	6.84E−07	PSMC4, PROCR, NAMPT, C1QBP, CANX, YWHAH, ACTN4, FUS, EIF2S1, SRI, FERMT3, AKR1B1, ARPC2, MAPRE2, CTNNA1, ARF4, PABPC1, ZYX, PPP1CA, RTN4, RPS2, RAC1, VCP, EZR, CSRP1, RPL5, YWHAB, ENG, RPL10A, HSPG2, KCTD12, ACTR2, CD99, YWHAQ, CD9, G3BP1, CTNND1, RPL6, PHB2
GO:0005885	Arp2/3 protein complex	0.004	ARPC2, ACTR2, ARPC4

## 4 Discussion

The complications arising from fluid resuscitation following hemorrhagic shock remain a significant challenge. In particular, the inflammatory state resulting in diminished endothelial barrier integrity, combined with a reduced plasma oncotic pressure when crystalloid fluids are used for resuscitation, contributes to microvascular leakage and edema formation [69]. These issues can be exacerbated when combined with acute alcohol intoxication [15]. Acute alcohol intoxication weakens host defense mechanisms, diminishes cellular responses to inflammatory challenges, and increases susceptibility to infections, all of which can lead to greater morbidity and mortality [42, 43, 70, 71]. One of the reasons for the worse outcomes is that alcohol intoxication directly causes increased microvascular permeability [14]. We have previously investigated adding S1P to crystalloid resuscitation fluids and found that this approach reduces microvascular leakage in rat models of HSR and AAI/HSR [15, 16]. In the current study, we extend these findings by showing that administration of ApoM, the major carrier of S1P in plasma, also ameliorates AAI/HSR‐induced microvascular leakage in both the presence and absence of S1P administration. More specifically, we present novel evidence that ApoM treatment enhances barrier function of cultured HUVEC, ameliorates alcohol‐induced disruption of HUVEC monolayer barrier function and loss of VE‐cadherin from cell–cell junctions, and causes a brief yet significant activation of Rac1. These are the first findings showing that ApoM ameliorates microvascular leakage in an in vivo injury model, with evidence supporting that ApoM has a direct impact on the microvascular barrier.

Previous studies have explored the functions of S1P/ApoM as an axis. ApoM is considered the main carrier of S1P, and the interaction between ApoM and S1P is important for S1P to interact with its receptors and exert its functions [31, 39, 72]. Our laboratory group previously found that S1P treatment can ameliorate microvascular leakage caused by combined AAI/HSR [15, 16]. Our original thought was that adding ApoM together with S1P in resuscitation fluids would provide a greater reduction in AAI/HSR‐induced microvascular leakage than S1P alone. Consistent with our previous findings [15], S1P improves microvascular leakage caused by AAI/HSR, enhances HUVEC barrier function, rescues the disruption of HUVEC monolayer barrier function, and depletion of VE‐Cadherin resulting from alcohol. However, we were surprised to find that ApoM alone exerted an almost identical effect as either S1P alone or ApoM+S1P. While the explanation for this is currently not clear, it is worth noting that this study is the first to identify that ApoM by itself plays an important role in microvascular hyperpermeability elicited by shock.

Another key finding in this study was that the small GTPase Rac1 was found to be bound in a complex with ApoM in cultured endothelial cells. The Ras superfamily of small (21–25 kDa) GTPases is subdivided into five branches, namely Rho, Ras, Rab, Ran, and Arf [73]. To date, 23 members of the Rho family GTPases and 36 Ras family members are known [73, 74]. Rho GTPases Rac1, RhoA, and Cdc42, as well as the Ras family GTPase Rap1, have been well characterized in the regulation of endothelial barrier properties [68]. Besides its role in the maintenance of barrier properties under resting conditions, evidence showed that activation of Rac1 appears to be a suitable approach to protect barrier functions under inflammatory conditions. More specifically, Rac1 promotes the formation and stabilization of VE‐Cadherin‐mediated cell–cell adhesion by acting downstream of several growth factors and signaling molecules [75, 76, 77]. In our present study, we found that ApoM activates Rac1 in HUVECs, which leads us to consider that the activation of Rac1 caused by ApoM likely enhances the VE‐Cadherin integrity and improves the endothelial cell barrier function. However, we do not rule out that other less characterized GTPases may also influence endothelial permeability, as was shown recently that the Rho family GTPase Wrch‐1 regulates tight junctions in epithelial cells [78]. In addition, the additive effect of S1P and ApoM on endothelial barrier function suggests that Rac1 may serve as a convergence point for the two different stimuli to exert their effects on the endothelial barrier. It is possible that S1P and ApoM work through distinct mechanisms to regulate endothelial barrier function. Thus, Rac1 or other potential small GTPases could be our targets for the future in this study.

In the current study, we demonstrate for the first time that ApoM protects against microvascular leakage when administered during resuscitation following acute alcohol intoxication and hemorrhagic shock. We also show that ApoM enhances the endothelial cell barrier function and rescues the depletion of VE‐Cadherin caused by alcohol. Furthermore, we provide evidence that ApoM activates the small GTPase, Rac1 in the endothelial cells. Collectively, the data suggest that ApoM ameliorates AAI/HSR‐induced microvascular leakage through enhancing VE‐Cadherin integrity and improving the endothelial barrier function afterwards, at least in part through its ability to activate Rac1.

## 5 Perspectives

The data in this study demonstrate that ApoM administration can reduce microvascular permeability in a rodent model of combined alcohol intoxication and hemorrhagic shock. The findings suggest that ApoM has endothelial barrier‐protective properties.

## Data Availability

Original datasets can be made available upon request.
